# Long-term outcomes of induction chemotherapy followed by chemoradiotherapy using volumetric-modulated arc therapy as an organ preservation approach in patients with stage IVA-B oropharyngeal or hypopharyngeal cancers

**DOI:** 10.1093/jrr/rraa033

**Published:** 2020-06-17

**Authors:** Katsumaro Kubo, Yuji Murakami, Masahiro Kenjo, Nobuki Imano, Yuki Takeuchi, Ikuno Nishibuchi, Tomoki Kimura, Daisuke Kawahara, Tsutomu Ueda, Sachio Takeno, Yasushi Nagata

**Affiliations:** 1 Department of Radiation Oncology, Hiroshima University Hospital, 1-2-3 Kasumi Minami-ku Hiroshima-shi, Hiroshima 734-8553, Japan; 2 Hiroshima High-Precision Radiotherapy Cancer Center, 2-2 Futabanosato Higashi-ku Hiroshima-shi, Hiroshima 732-0057, Japan; 3 Department of Otorhinolaryngology, Hiroshima University Hospital, 1-2-3 Kasumi Minami-ku Hiroshima-shi, Hiroshima 734-8553, Japan

**Keywords:** induction chemotherapy, oropharyngeal cancer, hypopharyngeal cancer, chemoradiotherapy, volumetric-modulated arc therapy

## Abstract

The present study aimed to analyze treatment outcomes after induction chemotherapy followed by chemoradiotherapy (CRT) using volumetric-modulated arc therapy (VMAT) in patients with stage IVA-B oropharyngeal cancer (OPC) or hypopharyngeal cancer (HPC), with long-term observation, including examination of larynx preservation. A total of 60 patients with stage IVA-B OPC or HPC, who underwent induction TPF chemotherapy (a combination regimen consisting of docetaxel, cisplatin, and 5-fluorouracil) followed by CRT using VMAT were analyzed. Overall survival (OS), progression-free survival (PFS), laryngoesophageal dysfunction-free survival (LEDFS), and locoregional control (LRC) were calculated and compared. Univariate and multivariate analyses were performed to determine statistical differences in OS and LEDFS. The median follow-up period at the time of evaluation was 61 months. Twenty-six (43%) patients had OPC and 34 (57%) had HPC. The 5-year OS, PFS, LEDFS, and LRC rates were 57%, 52%, 52%, and 68%, respectively. Response to TPF therapy was the only significant predictive factor of OS and LEDFS in multivariate analyses. Regarding long-term toxicities, grade ≥ 2 late toxicities accounted for 15%. No patients experienced grade ≥ 3 xerostomia, and 5% of all patients developed grade 3 dysphagia. With long-term observation, the OS, PFS, and LEDFS rates were relatively good, and the incidence of late toxicities was low. TPF followed by CRT using VMAT was feasible and more effective in those who responded to induction chemotherapy.

## Introduction

Radiation therapy is a well-established standard treatment for locally advanced head and neck squamous cell carcinomas, and induction chemotherapy followed by chemoradiotherapy (CRT) is one of several treatment options [1]. Although induction chemotherapy does not demonstrate a survival benefit compared with CRT alone [2], interest in the utility of induction chemotherapy persists for a few reasons including a reduction in the likelihood of distant metastases, and improvement of local regional control and organ preservation.

In Japan, several studies have investigated the feasibility and efficacy of induction chemotherapy followed by CRT for locally advanced head and neck carcinomas [3–8]. These studies reported good treatment outcomes after induction chemotherapy followed by CRT, including organ preservation. However, the follow-up duration was only 18–43.3 months; hence, the long-term outcome of this treatment strategy in the Japanese population remains unclear. Long-term observation is important to accurately assess treatment outcome(s) such as larynx preservation rate and swallowing function. In addition, current radiation techniques, such as intensity-modulated radiation therapy (IMRT) and image-guided radiation therapy, were not considered in previous randomized trials.

In our institution, induction chemotherapy followed by CRT has been indicated for stage IVA-B head and neck cancer from 2009. Therefore, in the present study, we analyzed treatment outcomes after induction chemotherapy followed by CRT using volumetric-modulated arc therapy (VMAT) in patients with stage IVA-B oropharyngeal cancer (OPC) or hypopharyngeal cancer (HPC) with long-term observation, including an examination of laryngeal dysfunction.

## Materials and methods

### Patients

Data from patients, who were diagnosed with locally advanced OPC or HPC and underwent definitive radiation therapy (RT) at Hiroshima University Hospital (Hiroshima, Japan) between July 2009 and June 2017, were retrospectively analyzed. Individuals who fulfilled the following criteria were included in the present study: histological diagnosis of squamous cell carcinoma of the oropharynx or hypopharynx; stage IVA-B disease and no distant metastasis; underwent induction chemotherapy consisting of a combination regimen of docetaxel, cisplatin, and 5-fluorouracil (i.e., TPF therapy); and definitive CRT using VMAT. Patients with a follow-up duration < 2 years without death were excluded from this study.

Before induction chemotherapy, all patients underwent clinical evaluation including assessment of medical history, physical examinations and laboratory investigations, endoscopy, ultrasound examination, and radiographic studies. Based on the above information, we discuss the treatment of each head and neck cancer patient on the cancer board by multidisciplinary team for head and neck cancer. Laboratory investigations included complete blood cell count, liver function, renal function, and measurement of electrolyte levels. Radiographic studies included contrast-enhanced computed tomography (CT), ^18^F-fluorodeoxyglucose positron emission tomography-CT (PET/CT), and magnetic resonance imaging (MRI). Almost 60% of patients with OPC did not undergo human papillomavirus (HPV) status testing. The clinical TNM stage was defined according to the Tumor Node Metastasis classification (Union for International Cancer Control, 7th Edition). The study was approved by the Human Ethics Review Committee of Hiroshima University Hospital.

### Treatment

#### Radiation therapy

Before treatment, the heads of all patients were non-invasively immobilized using a thermoplastic head-neck-shoulder mask, and subjected to contrast-enhanced CT. CT images were acquired at a slice thickness of 2.5 mm and imported to the Eclipse treatment planning system (Varian Medical Systems, Palo Alto, CA, USA) for VMAT planning (Rapidarc; Varian Medical Systems, Palo Alto, CA, USA). Gross tumor volume (GTV) was based on clinical, endoscopic, and radiological findings according to CT, MRI, and PET/CT. GTV was determined based on radiological findings before induction chemotherapy. Clinical target volume (CTV) 70 was generated by adding a 5–10 mm margin from GTV, including the primary tumor and involved lymph nodes. CTV63 included the high-risk areas and was created individually based on primary tumor site and lymph node metastases. CTV56 included elective nodal regions. Planning target volume (PTV) 70, PTV63, and PTV56 were generated by adding a 5–10 mm margin from CTV70, CTV63, and CTV56. Usually, 2 or 3 axial coplanar arcs were used for VMAT. All treatment plans were designed based on a TrueBeam linear accelerator equipped with 5 mm leaf-width multi-leaf collimators (Varian Medical Systems, Palo Alto, CA, USA). All plans were normalized so that PTV70 D95 (the dose that covers 95% of the PTV70) was equal to 70 Gy using 6–10 MV photon beams. PTV70, PTV63, and PTV56 received a total dose of 70 Gy, 63 Gy, and 56 Gy in 35 fractions, respectively. Cone-beam CT was performed daily for patient set-up and positioning verification.

#### Chemotherapy

All patients underwent induction chemotherapy followed by CRT. The medication administered was TPF, a combination chemotherapy regimen consisting of docetaxel (70 mg/m^2^) on day 1, cisplatin (70 mg/m^2^) on day 4, and 5-fluorouracil (750 mg/m^2^) by 24 h continuous infusion for 5 days. Two cycles at 28-day intervals were planned.

The concurrent chemotherapy regimen was 3 cycles of cisplatin 100 mg/m^2^ at an interval of 3 weeks. If the administration of cisplatin was not suitable due to patient general condition, renal function, or age, carboplatin or cetuximab was administered.

### Statistical analysis

The Kaplan-Meier method was used to calculate overall survival (OS), progression-free survival (PFS), laryngoesophageal dysfunction-free survival (LEDFS), and locoregional control (LRC). OS was defined as the date of initiation of CRT to the date of the final follow-up or death from any cause. PFS was defined as the date of initiation of CRT to the date of any tumor progression or death from any cause. LRC was estimated as the CRT start date until the date of any locoregional progression; patients without locoregional progression at the time of death were censored. In estimating LRC and PFS, primary tumor progression was defined as enlargement of the tumor from its state after CRT, as observed by CT or endoscopic findings, even in patients who did not achieve complete response following CRT. In patients in whom the tumor remained evident after CRT and underwent subsequent surgery in which the residual tumor was pathologically assessed, it was included as an event of local progression. LEDFS was analyzed to evaluate organ preservation in the cohort. For LEDFS, the Larynx Preservation Consensus Panel recommends including death, local recurrence, total or partial laryngectomy, tracheotomy at ≥2 years, or feeding tube at ≥2 years [9]. Swallowing function was evaluated with videofluorography. Response to induction chemotherapy was divided into two groups according to the Response Evaluation Criteria in Solid Tumors: responder and non-responder [10]. A responder was defined that both the primary tumor and involved lymph nodes showed a partial or complete response. A non-responder was defined that the primary tumor and/or involved lymph nodes showed stable or progressive disease. The χ2 test was conducted to determine the significant differences between responders and non-responders. Univariate analyses using the Cochran-Mantel-Haenszel test and multivariate analyses using the Cox proportional hazards model were performed to compare and determine statistical differences in OS and LEDFS. BellCurve version 3.20 (Social Survey Research Information Co., Ltd., Tokyo, Japan) for Excel (Microsoft Corporation, Redmond, WA, USA) was used to perform statistical analyses. Differences with *p* < 0.05 were considered to be statistically significant. Treatment-related toxicities were evaluated according to the Common Terminology Criteria for Adverse Events ver 4.0.

## Results

### Patients

A total of 60 eligible patients, the characteristics of whom are summarized in Table 1, were enrolled in the present study. The median age of the patients was 65 years (range, 36–80 years) and 91.7% were men. Twenty-six (43.3%) patients had OPC and 34 (56.7%) had HPC. Of the 26 patients with OPC, HPV infection status was analyzed in 10, of whom 5 were found to be HPV positive. T1, T2, T3, T4a, and T4b disease were observed in 4 (6.7%), 18 (30.0%), 7 (11.7%), 22 (36.7%), and 9 (15.0%) patients, respectively. Regarding lymph node metastases, N0, N1, N2, and N3 disease were observed in 6 (10.0%), 2 (3.3%), 49 (81.7%), and 3 (5.0%) patients, respectively. Regarding response to TPF therapy, 48 (80.0%) patients were responders, and 12 (20.0%) were non-responders. Of the 12 non-responders, there were 12 stable disease cases and no progressive disease case after induction TPF chemotherapy, but all refused surgery. Table 2 shows the characteristics of responders and non-responders. The responders had relatively early stage disease compared to non-responders (*p* = 0.0359). Cisplatin was administered to 55 (91.7%) patients, cisplatin plus 5-fluorouracil to 3 (5.0%), cetuximab to 1 (1.7%), and carboplatin to 1 (1.7%) with RT. The median follow-up at the time of evaluation was 60.5 months (range, 28–92 months) in survivors.

**Table 1:** Patient characteristics

	N = 60	100 (%)
Age, years, median (range)	65 (36–80)	−
Sex		
Male	55	91.7
Female	5	8.3
Location		
Hypopharynx	34	56.7
Postcricoid area	2	3.3
Piriform sinus	24	40
Posterior pharyngeal wall	8	13.3
Oropharynx	26	43.3
Anterior wall	9	15
Lateral wall	13	21.7
Posterior wall	2	3.3
Superior wall	2	3.3
HPV status		
HPV-positive	5	8.3
HPV-negative	5	8.3
Unknown	16	26.7
T category		
T1	4	6.7
T2	18	30
T3	7	11.7
T4a	22	36.7
T4b	9	15
N category		
N0	6	10
N1	2	3.3
N2a	1	1.7
N2b	41	68.3
N2c	7	11.7
N3	3	5
Clinical stage		
IVA	48	80
IVB	12	20
PET-CT Yes	57	95
Response to TPF		
Responder	48	80
Non-responder	12	20
Concurrent chemotherapy		
Cisplatin	55	91.7
Cisplatin +5-fluorouracil	3	5
Cetuximab	1	1.7
Carboplatin	1	1.7

HPV, Human Papillomavirus; PET-CT, 18F-fluorodeoxyglucose positron emission tomography computed tomography; TPF, combination chemotherapy regimen consisting of docetaxel, cisplatin, and 5-fluorouracil

**Table 2:** Characteristics of responders and non-responders

	Responder		Non-responder		*p* value
	N = 48	100(%)	N = 12	100(%)	
Age					0.4383
≤ 65	26	54.2	5	41.7	
> 65	22	45.8	7	58.3	
Sex					0.5593
Male	43	89.6	12	100	
Female	5	10.4	0	0	
Location					0.6023
Oropharynx	20	41.7	6	50.0	
Hypopharynx	28	58.3	6	50.0	
Clinical stage					0.0359
IVA	41	85.4	7	58.3	
IVB	7	14.6	5	41.7	
T category					0.6054
T1–3	24	50.0	5	41.7	
T4	24	50.0	7	58.3	
N category					1.0000
N0–1	6	12.5	2	16.7	
N2–3	42	87.5	10	83.3	
Vocal cord impairment					0.5593
Yes	10	20.8	1	8.3	
No	38	79.2	11	91.7	
Smoking history					1.0000
Yes	37	77.1	9	75.0	
No	11	22.9	3	25.0	
Course of cisplatin concurrent with RT^*^					0.9493
1 or 2	22	48.9	5	50	
3	23	51.1	5	50	

RT, radiation therapy

^*^Five patients did not receive cisplatin concurrent with RT, and 45 responders and 10 non-responders received cisplatin with RT.

### Treatment outcomes

**Fig. 1.**
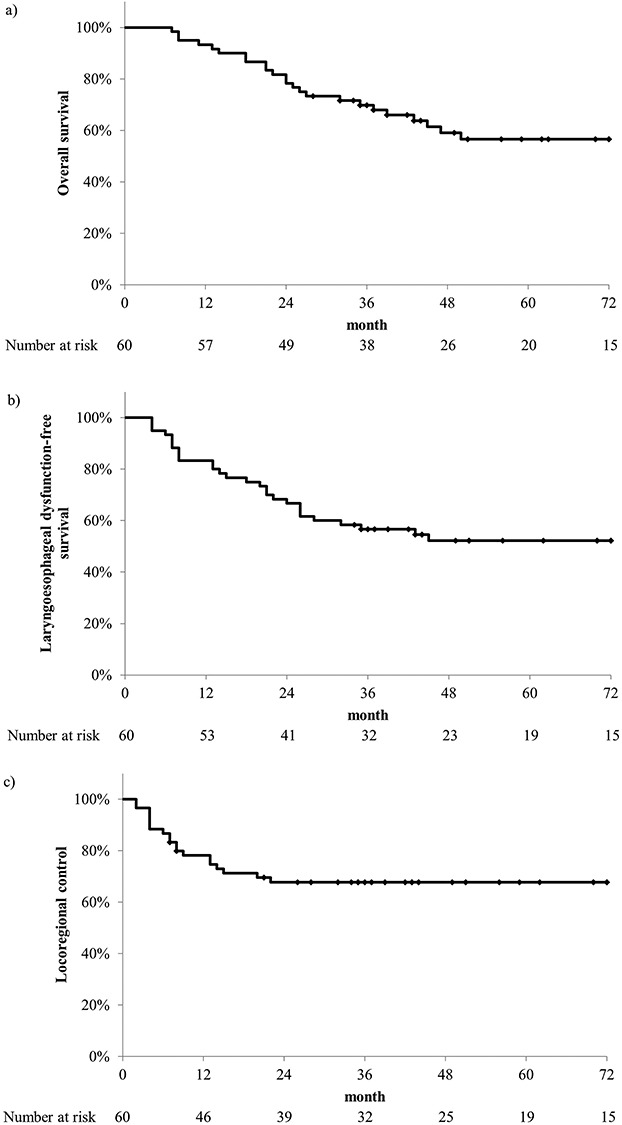
Overall survival (OS), laryngoesophageal dysfunction-free survival (LEDFS), and loco-regional control (LRC) in all patients. a) The 5-year OS rate was 56.6%. b) The 5-year LEDFS rate was 52.2%. c) The 5-year LRC rate was 67.7%.

OS, LEDFS, and LRC rates in all patients are presented in Figure 1. At the last follow-up, 34 (56.7%) patients were still alive, 23 (38.3%) died from the disease, and 3 (5.0%) died of other causes. The 5-year OS rate was 56.6% (95% confidence interval [CI] 43.2–70.1%). The 5-year OS rates in those with OPC and HPC were 60.4% (95% CI 41.0–79.7%) and 53.6% (95% CI 35.1–72.2%), respectively. The 5-year LEDFS rate was 52.2% (95% CI 39.1–65.2%). The 5-year LEDFS rates in those with OPC and HPC were 53.9% (95% CI 34.7–73.0%) and 50.3% (95% CI 32.4–68.2%), respectively. Events associated with laryngoesophageal dysfunction included death in 14 patients, local recurrence in 13, and feeding tube at ≥2 years in 3. Of the 3 patients depending on feeding tube, one could not eat before treatment according to the tumor spread, and needed feeding tube until the end of observation period.

The 5-year LRC rate was 67.7% (95% CI 55.8–79.7%). The 5-year LRC rates in those with OPC and HPC were 60.7% (95% CI 41.7–79.8%) and 73.1% (95% CI 58.1–88.2%), respectively.

### Predictive factors of overall survival and laryngoesophageal dysfunction-free survival

**Fig. 2.**
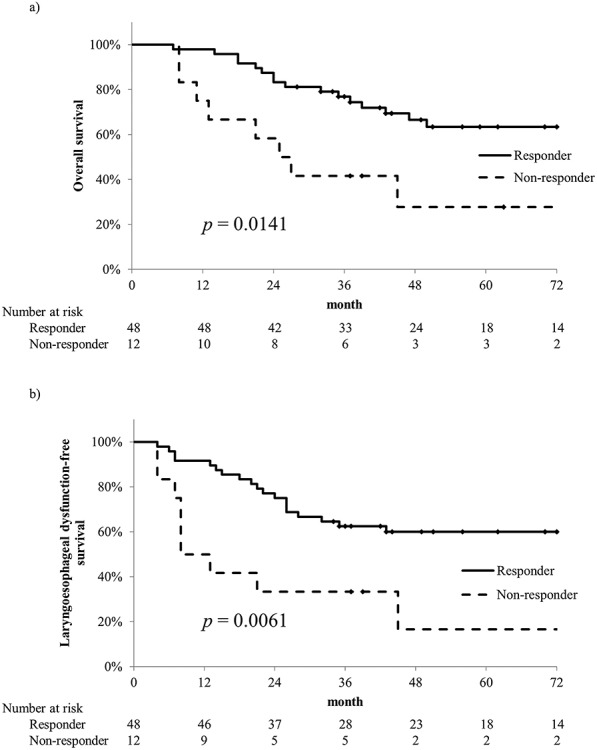
Overall survival (OS) and laryngoesophageal dysfunction-free survival (LEDFS) in responders and non-responders to induction chemotherapy. a) The 5-year OS rates of responders and non-responders were 63.5% and 27.8%, respectively. b) The 5-year LEDFS rates of responders and non-responders were 59.9% and 16.7%, respectively.

Results of univariate and multivariate analyses of OS and LEDFS are summarized in Table 3. Response to TPF therapy was the only significant predictive factor of OS and LEDFS in the univariate analysis, and remained significant in the multivariate analysis. The OS and LEDFS rates of responders and non-responders are presented in Figure 2.

**Table 3:** Univariate and multivariate analysis of predictive factors

	Overall survival			Laryngoesophageal dysfunction-free survival		
Investigated factors	UVA	MVA	Hazard ratio (95% CI)	UVA	MVA	Hazard ratio (95% CI)
	*p* value	*p* value		*p* value	*p* value	
Age (≤65 vs > 65)	0.2717	ns	−	0.2802	ns	−
Sex (male vs female)	0.5934	ns	−	0.6998	ns	−
Location (oropharynx vs hypopharynx)	0.8324	ns	−	0.9191	ns	−
Clinical stage (IVA vs IVB)	0.3464	ns	−	0.6385	ns	−
T category (T1–3 vs T4)	0.3586	ns	−	0.4706	ns	−
N category (N0–1 vs N2–3)	0.4187	ns	−	0.7904	ns	−
Response to TPF (responder vs non-responder)	0.0141	0.0189	0.3653 (0.1576–0.8466)	0.0061	0.0094	0.3508 (0.1591–0.7737)

UVA, univariate analysis; MVA, multivariate analysis; CI, confidence interval; ns, nonsignificant; TPF, combination chemotherapy regimen consisting of docetaxel, cisplatin, and 5-fluorouracil

### Recurrence patterns

The 5-year PFS rate was 51.6% (95% CI 39.0–64.3%). The 5-year PFS rates in those with OPC and HPC were 57.7% (95% CI 38.7–76.7%) and 46.9% (95% CI 30.0–63.7%), respectively.

Disease progression was observed in 28 (46.7%) patients. As the pattern of first recurrence, 18 (30.0%) patients experienced treatment failure at the site of the primary tumor and/or involved lymph node, and 9 (15.0%) developed distant metastases. Of the 28 patients, only 1 (1.7%) experienced regional lymph node recurrence excluding the involved lymph node at diagnosis as the first recurrence. Overall, during the long-term observation period, 18 (30.0%) patients experienced primary tumor and/or involved lymph node recurrence, 15 (25.0%) developed distant metastases, and 3 (5.0%) experienced regional lymph node recurrence.

### Toxicity

Acute and late toxicities are listed in Table 4. During CRT, the most common grade 3 toxicity was mucositis (36.7%), followed by dysphagia (31.7%), and dermatitis (25.0%). Overall, grade 3 non-hematological toxicities were observed in 34 (56.7%) patients. Regarding late toxicities, grade 2 dysphagia was not observed, grade 3 dysphagia was observed in 3 (5.0%) patients, grade 2 xerostomia in 3 (5.0%), and grade 2 dysgeusia in 3 (5.0%). Grade 2 osteonecrosis of the jaw was noted in 2 (3.3%) patients. Therefore, 9 (15.0%) patients developed grade ≥ 2 late toxicity.

**Table 4:** Acute and late toxicities

Toxicity	Common Terminology Criteria for Adverse Events ver 4.0					
	Grade 0–1		Grade 2		Grade 3	
	N	100 (%)	N	100 (%)	N	100 (%)
Acute toxicity						
Dysphagia	9	15.0	32	53.3	19	31.7
Mucositis	5	8.3	33	55.0	22	36.7
Dermatitis	23	38.3	22	36.7	15	25.0
Dysgeusia	30	50.0	30	50.0	0	0
Xerostomia	31	51.7	29	48.3	0	0
Late toxicity						
Dysphagia	57	95.0	0	0	3	5.0
Dysgeusia	57	95.0	3	5.0	0	0
Xerostomia	57	95.0	3	5.0	0	0
Osteonecrosis of jaw	0	0	2	3.3	0	0

## Discussion

Concurrent CRT is regarded to be the standard treatment option for patients with locally advanced head and neck cancers and one of several organ preservation strategies [1, 2]. However, this intensification of treatment has resulted in increased short- and late-term adverse events, with a previous study reporting that almost 50% of patients with locally advanced head and neck cancers treated with concurrent CRT experienced long-term dysphagia [11]. In addition, the long-term results of the Radiation Therapy Oncology Group (RTOG) 91–11 trial demonstrated an unexplained increase in deaths unrelated to cancer in patients who underwent concurrent CRT, and indicated that laryngeal dysfunction may be associated with death [2]. Therefore, the preservation of larynx morphology and function is an important focus of contemporary studies, and the Larynx Preservation Consensus Panel has recommended LEDFS as a new endpoint. Induction chemotherapy is expected to improve larynx preservation. In Japan, several studies have described treatment outcomes after induction chemotherapy followed by CRT, including organ preservation [3–8]. However, the follow-up duration was relatively short. Long-term observation is important to accurately assess laryngeal dysfunction. Although long-term results of previous randomized trials have been reported, these studies lacked the examination of LEDFS and current radiation techniques such as IMRT [12, 13]. Therefore, long-term outcomes, including LEDFS, of induction chemotherapy followed by CRT using current radiation techniques remain unclear. To address this concern, we analyzed long-term outcomes after induction chemotherapy followed by CRT using current radiation techniques in patients with stage IVA-B OPC or HPC. In our study, the median follow-up period was 61 months. The 5-year OS, PFS, LEDFS, and LRC rates were 57%, 52%, 52%, and 68%, respectively.

The clinical results of previous studies investigating CRT for locally advanced head and neck cancers are shown in Table 5. Although most of these studies included stage III head and neck cancers, our study included stage IV disease [7, 12–15]; nevertheless, our results demonstrated similar efficacy. Regarding larynx preservation, few studies have addressed LEDFS after CRT in patients with locally advanced OPC or HPC with long-term observation. The 5-year LEDFS rate in laryngeal-preservation protocols for locally advanced laryngeal cancer was 30–45% [16, 17]. The 5-year LEDFS rate in the present study was 52% and was relatively good, even with long-term observation despite the fact that all patients had stage IV disease.

**Table 5:** Clinical results reported in other studies

	N	median follow-up (month)	Site	Stage	Treatment	5-year survival rate (%)		
						OS	PFS	LEDFS
This study	60	61	Oropharynx Hypopharynx	IV	TPF- > CRT	57	52	52
EORTC 24954^14^	224	78	Hypopharynx Larynx	II-IV	FP- > RT	49	41	31^*^
TAX 324^12^	255	72	Oropharynx Hypopharynx Larynx Other	III/IV	TPF- > CRT	52	45	NA
GORTEC 2000–01^13^	110	105	Hypopharynx Larynx	III/IV	TPF- > CRT	51	42	67^**^
						3-year survival rate (%)		
This study	60	61	Oropharynx Hypopharynx	IV	TPF- > CRT	70	52	57
Caudell et al^15^	85	50	Hypopharynx Larynx	III/IV	CRT	49	61	29
ECRIPS^7^	54	36	Oropharynx Hypopharynx Larynx	III/IV	TPEx- > BRT	91	58	60

OS, overall survival; PFS, progression-free survival; LEDFS, laryngoesophageal dysfunction-free survival; TPF, combination chemotherapy regimen consisting of docetaxel, cisplatin, and 5-fluorouracil; CRT, chemoradiotherapy; FP, combination chemotherapy regimen consisting of cisplatin and 5-fluorouracil; TPEx, combination chemotherapy regimen consisting of docetaxel, cisplatin, and cetuximab; BRT, bioradiotherapy

^*^5-year survival with a functional larynx

^**^5-year larynx dysfunction-free survival

In this study, response to TPF therapy was the only significant predictive factor of OS and LEDFS. Several previous studies have reported that response to induction chemotherapy may be a predictive factor of treatment outcome after CRT for locally advanced head and neck cancers [18]. Accordingly, Gorphe et al. altered the treatment strategy after induction chemotherapy depending on response [16]. Our results also supported their investigations, and we believe that non-responders to induction chemotherapy should undergo surgery. On the other hand, age, sex, and TNM stage, which have been reported to be correlated with survival, were not significant in this study [19–21], although this could simply be explained by the small sample size.

We also assessed long-term toxicities. Grade ≥ 2 late toxicities accounted for only 15%, and most other patients experienced asymptomatic or grade 1 toxicities. In the GORTEC 2000–01 trial, grade ≥ 3 late toxicities of the salivary gland accounted for 7% in the TPF arm [13]. In the long-term results of the TAX324 trial, 3% of patients in the TPF treatment arm were dependent on a gastric feeding tube [12]. In our study, no patients experienced grade ≥ 3 xerostomia and grade 2 dysphagia, and only 5% of all patients developed grade 3 dysphagia and required a gastric feeding tube. The incidence of late toxicities in our study were acceptable and lower than that in the RTOG 91–11 trial not using IMRT [2]. Tumor shrinkage by induction chemotherapy and radiation dose reduction in VMAT for organ at risk may have contributed to this result. In addition, deaths unrelated to cancer in this study were only 3 cases, and also lower than those of RTOG 91–11. Since it was indicated that laryngeal dysfunction may be associated with deaths unrelated to cancer, induction chemotherapy followed by CRT using VMAT might contribute to the reduction of the deaths and to the improvement of OS.

The present study had several limitations, including its retrospective single-center design, and the fact that almost 60% of patients with OPC did not undergo HPV status testing and 5 with HPV-positive OPC were included. This is because HPV status testing for OPC is currently not covered by insurance in Japan. In our institution, HPV status testing has been routinely performed since 2014. These facts introduced potential biases. Nevertheless, this study was meaningful in reporting long-term outcomes after induction chemotherapy followed by CRT using current radiation techniques in patients with locally advanced OPC or HPC.

In conclusion, with long-term observation, the OS, PFS, and LEDFS rates in patients with stage IVA-B OPC or HPC after induction chemotherapy followed by CRT using VMAT were relatively good, and the incidence of late toxicities was low. In addition, response to TPF therapy was the only significant predictive factor of OS and LEDFS. CRT using VMAT was more effective in those who responded to induction chemotherapy.

## Conflicts of interest

None.
